# Kidney Replacement Therapy in COVID-19 Induced Kidney Failure and Septic Shock: A Pediatric Continuous Renal Replacement Therapy [PCRRT] Position on Emergency Preparedness With Resource Allocation

**DOI:** 10.3389/fped.2020.00413

**Published:** 2020-07-03

**Authors:** Rupesh Raina, Ronith Chakraborty, Sidharth Kumar Sethi, Timothy Bunchman

**Affiliations:** ^1^Department of Nephrology, Cleveland Clinic Akron General, Akron Nephrology Associates, Akron, OH, United States; ^2^Department of Nephrology, Akron Children's Hospital, Akron, OH, United States; ^3^Pediatric Nephrology & Pediatric Kidney Transplantation, Kidney and Urology Institute, Medanta, The Medicity Hospital, Gurgaon, India; ^4^Pediatric Nephrology & Transplantation, Children's Hospital of Richmond, Virginia Commonwealth University, Richmond, VA, United States

**Keywords:** COVID-19, extracorporeal therapy, kidney replacement therapy, pediatrics, acute kidney injury

## Abstract

The recent worldwide pandemic of COVID-19 has had a detrimental worldwide impact on people of all ages. Although data from China and the United States indicate that pediatric cases often have a mild course and are less severe in comparison to adults, there have been several cases of kidney failure and multisystem inflammatory syndrome reported. As such, we believe that the world should be prepared if the severity of cases begins to further increase within the pediatric population. Therefore, we provide here a position paper centered on emergency preparation with resource allocation for critical COVID-19 cases within the pediatric population, specifically where renal conditions worsen due to the onset of AKI.

## Introduction

The recent worldwide pandemic of COVID-19, also known as severe acute respiratory syndrome coronavirus 2 (SARS-CoV-2), has led CoVs to become one of the major pathogens of evolving respiratory disease outbreaks (1). Overall, up to 26% of hospitalized adults have been reported to require support in an intensive care unit (ICU) due to acute respiratory distress syndrome (ARDS) and multiple organ dysfunction/failure (MOD/MOF) (2–4). More specifically, acute kidney injury (AKI) has recently been reported by various epidemiological and clinical characteristics studies, demonstrating the presence of AKI symptoms in 3–23% of COVID-19 patients (2, 4–11).

**Figure 1:**
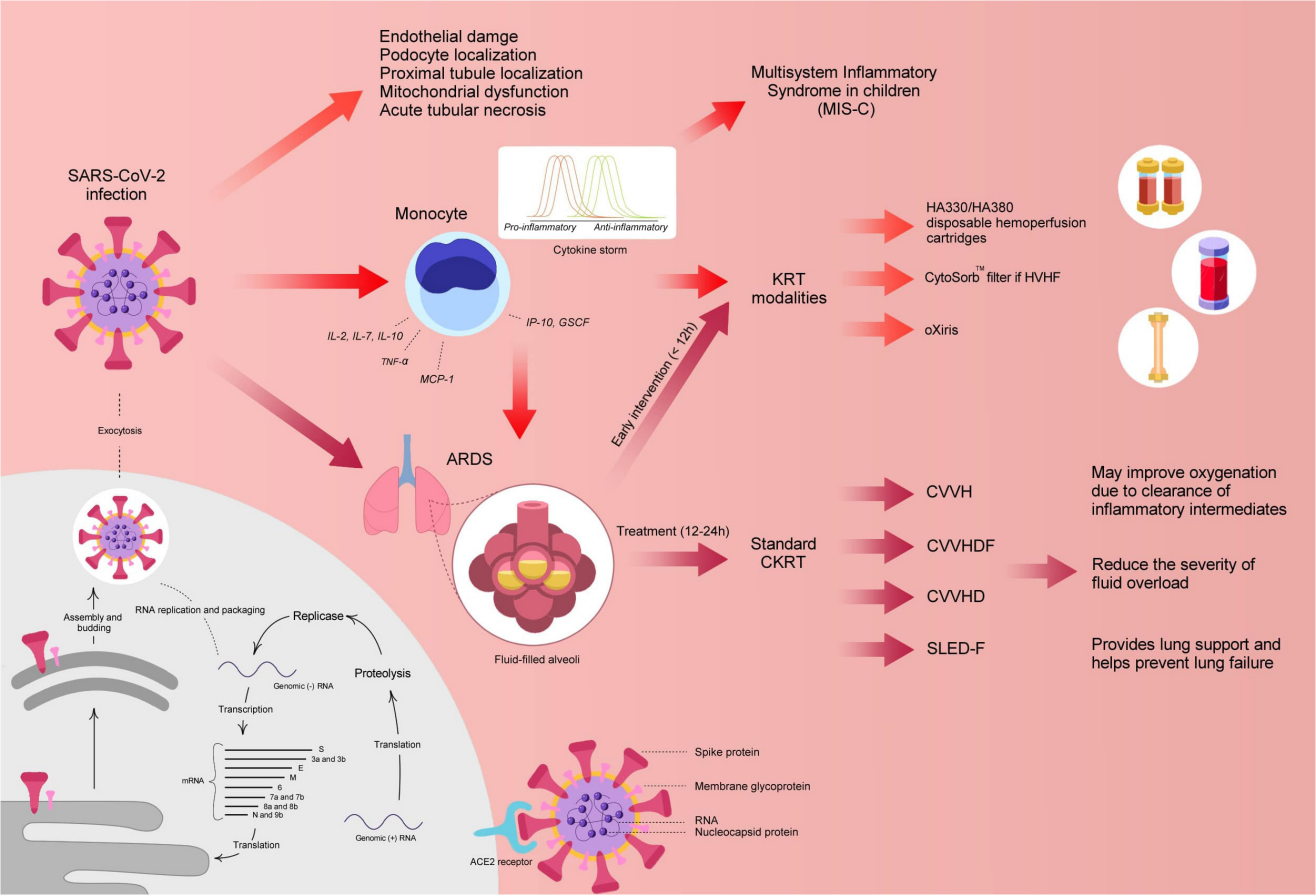
Potential mechanism of COVID-19 and postulated treatments. ACE2, angiotensin-converting enzyme 2; ARDS, acute respiratory distress syndrome; CKRT, continuous kidney replacement therapy; CVVH, continuous venovenous hemofiltration; CVVHD, continuous venovenous hemodialysis; CVVHDF, continuous venovenous hemodiafiltration; GSCF, granulocyte-colony stimulating factor; IL, interleukin; IP-10, Interferon-inducing protein-10; KRT, kidney replacement therapy; MCP, monocyte chemoattractant protein 1; RNA, ribonucleic acid; SLEDD-f, sustained low-efficiency daily diafiltration; TNF-α, tumor necrosis factor-alpha. Designed and created by Joshua Colina, joshcolina@gmail.com.

Currently, the exact mechanism of kidney involvement in COVID-19 patients is unclear; although, various mechanisms have been postulated including virus-induced cytopathy of renal tissue and sepsis due to cytokine storm syndrome (Figure 1). Similar to other CoVs, the spike (S) glycoprotein of the COVID-19 virus binds angiotensin converting enzyme 2 (ACE2) receptors on host cells (12, 13). Afterwards, the active S protein is cleaved by transmembrane serine proteases (TMPRSSs) resulting in membrane fusion facilitated by fusion peptides released by the virus. This mechanism implies that ACE2 and TMPRSSs are key factors in the infection of host cells (12, 13).

In relation to kidney involvement, a recent study by Xu et al. reported that ACE2 and TMPRSS2 genes were co-expressed significantly in podocytes and proximal convoluted tubules, similar to that in the lung, small intestine, and colon (14). The podocytes are highly specialized cells of the kidney glomerulus and are quite susceptible to viral and bacterial infections. Injury to the structure of podocytes can induce a massive leak of proteins into the urine. In an ongoing case study by Li et al., the authors reported that 34% of COVID-19 infected adults acquired heavy proteinuria on the first day of admission with 63% presenting with proteinuria during their hospital stay (15). The authors suggested that renal impairment may have been a result of the COVID-19 induced cytopathy of podocytes and renal tubular cells (15).

Furthermore, cytokine storm syndrome has been postulated as another mechanism in causing MOD, including renal function impairment. Various studies have suggested the occurrence of cytokine storm in critically ill COVID-19 patients due to higher plasma circulating cytokine levels (interleukin [IL]-2, IL-7, IL-10, interferon-inducing protein-10 [IP-10], granulocyte-colony stimulating factor [GSCF], macrophage inflammatory protein-1a [MIP1a], monocyte chemoattractant protein 1 [MCP1], and tumor necrosis factor alpha [TNF-α]) in severe cases (2). The cytokine storm can cause sepsis and lead the immune system to target normal cells, such as podocytes in the lung and kidney, in an effort to eradicate the virus (14). In addition, it has been postulated that viral involvement and the hosts inflammatory response leads to the induction of endothelial dysfunction, causing organ dysfunction in a variety of organ systems (16) (Figure 1).

However, the epidemiology and characteristics of COVID-19 have been reported to be much different in the pediatric population (<18 years). In comparison to adults, the incidence of COVID-19 in the pediatric subset ranges from 0.8 to 2.7% with 0.58–9.7% of patients being admitted to the ICU (17–20) (Table 1). Additionally, data from China and the United States show that pediatric cases often have a mild course and are less severe than in adults (17, 20). In a study by Dong et al. of 2,143 COVID-19 pediatric patients, it was reported that 94.1% of cases were either asymptomatic (4.4%), mild (50.9%), or moderate (38.8%), suggesting that clinical manifestations in children were less severe compared with adults (20).

**Table 1:** Epidemiology of COVID-19 pediatric patients.

**Study**	**Location**	**Total cases**	**Incidence (*n*, %)**	**Admitted to ICU**
CDC (17)	United States	149,760	2,572 (1.7%)	15 (0.58%)
Livingston and Bucher (18)	Italy	22,512	270 (1.2%)	N/A
Tagarro et al. (19)	Spain (Madrid)	4,6,95	41 (0.8%)	4 (9.7%)
Dong et al. (20)	China	80,174	2,143 (2.7%)	13 (0.6%)
VPS (21)	North America	9,186	401 (4.4%)	401 (100%)*

*ICU, intensive care unit*.

* *This data is from pediatric ICUs so all patients were admitted to the ICU*.

Nevertheless, several cases of multisystem inflammatory syndrome (MIS-C) have recently been reported in pediatric COVID-19 patients (21–25). Patients with MIS-C presented with various symptoms including a persistent fever, hypotension, elevated inflammatory markers, and multiorgan involvement (22). As of May 12, 2020, a 102 cases of MIS-C had been reported in pediatric COVID-19 patients in the state of New York (22). In the United Kingdom, eight cases of MIS-C were reported during a period of 10 days in mid-April (Table 2) (23, 24). One out of these eight pediatric patients required KRT and ECMO and died (23, 24). Additionally, according to the Virtual Pediatric Systems (VPS), a total of 218 cases of MIS-C in pediatric patients (<18 years) with seven deaths have been reported throughout pediatric ICUs in North America (21). However, there is still currently limited information regarding the pathogenesis, risk factors, and treatment for MIS-C and it remains unknown whether this condition is specific to the pediatric population.

**Table 2:** Patients exhibiting multisystem inflammatory syndrome.

**Patient**	**Characteristics**	**Clinical presentation**	**Support modality**	**Treatment**	**Lab results**	**Microbiology status**	**Outcome (PICU LOS; status)**
1	Male, 14 years, Afro-Caribbean.	Fever: 4 d >40°C. Diarrhea; abdominal pain; headache	MV, KRT, VA-ECMO	Dopamine, noradrenaline, argipressin, adrenaline, milrinone, hydrocortisone, IVIG, ceftriaxone, lindamycin	Ferritin 4,220 μg/L; D-dimers 13·4 mg/L; troponin 675 ng/L; proBNP >35,000; CRP 556 mg/L; procalcitonin>100 μg/L; albumin 20 g/L; platelets 123 × 10^9^	SARS-CoV-2 positive (post-mortem)	6 days; deceased due to right MCA and ACA ischemic infarction.
2	Male, 8 years, Afro-Caribbean.	Fever: 5 d >39°C. Diarrhea; abdominal pain; conjunctivitis; rash	MV	Noradrenaline, adrenaline, IVIG, infliximab, methylprednisolone, ceftriaxone, lindamycin	Ferritin 277 μg/L; D-dimers 4·8 mg/L; troponin 25 ng/L; CRP 295 mg/L; procalcitonin 8·4 μg/L; albumin 18 g/L; Platelets 61 × 10^9^	SARS-CoV-2 negative (likely exposure from mother)	4 days; alive
3	Male, 4 years, Middle Eastern.	Fever: 4 d >39°C. Diarrhea; vomiting; abdominal pain; conjunctivitis	MV	Noradrenaline, adrenaline, IVIG ceftriaxone, clindamycin	Ferritin 574 μg/L; D-dimers 11·7 mg/L; troponin 45 ng/L; CRP 322 mg/L; procalcitonin 10·3 μg/L; albumin 22 g/L; Platelets 103 × 10^9^	Adenovirus positive; HERV positive	4 days; alive
4	Female, 13 years, Afro-Caribbean.	Fever: 5 d >39°C. Diarrhea; abdominal pain; conjunctivitis	HFNC	Noradrenaline, milrinone, IVIG,ceftriaxone, lindamycin	Ferritin 631 μg/L; D-dimers 3·4 mg/L; troponin 250 ng/L; proBNP 13,427 ng/L; CRP 307 mg/L; procalcitonin 12·1 μg/L; albumin 21 g/L; Platelets 146 × 10^9^	SARS-CoV-2 negative	5 days; alive
5	Male, 6 years, Asian.	Fever: 4 d >39°C. Odynophagia; conjunctivitis; rash	NIV	Milrinone, IVIG, methylprednisolone, aspirin, ceftriaxone	Ferritin 550 μg/L; D-dimers 11·1 mg/L; troponin 47 ng/L; NT-proBNP 7,004 ng/L; CRP 183 mg/L; albumin 24 g/L; platelets 165 × 10^9^	SARS-CoV-2 positive (likely exposure from father)	4 days; alive
6	Female, 6 years, Afro-Caribbean.	Fever: 5 d >39°C. Diarrhea & vomiting (3 d); myalgia; conjunctivitis	NIV	Dopamine, noradrenaline, milrinone, IVIG, methylprednisolone, aspirin, ceftriaxone, clindamycin	Ferritin 1,023 μg/L; D-dimers 9·9 mg/L; troponin 45 ng/L; NT-proBNP 9,376 ng/L; CRP mg/L 169; procalcitonin 11·6 μg/L; albumin 25 g/L; platelets 158	SARS-CoV-2 negative (likely exposure from grandfather)	3 days; alive
7	Male, 12 years, Afro-Caribbean.	Fever: 4 d >39°C. Diarrhea & vomiting (2 d); abdominal pain; headache; rash; odynophagia	MV	Noradrenaline, adrenaline, milrinone, IVIG, methylprednisolone, heparin, ceftriaxone, clindamycin, metronidazole	Ferritin 958 μg/L; D-dimer 24·5 mg/L; troponin 813 ng/L; NT-proBNP >35 000 ng/L; CRP 251 mg/L; procalcitonin 71·5 μg/L; Albumin 24 g/L; Platelets 273 × 10^9^	SARS-CoV-2 negative	4 days; alive
8	Female, 8 years, Afro-Caribbean.	Fever: 4 d >39°C. Diarrhea & vomiting (2 d); abdominal pain; odynophagia	MV	Dopamine, noradrenaline, milrinone, IVIG, aspirin, ceftriaxone, clindamycin	Ferritin 460 μg/L; D-dimers 4·3 mg/L; troponin 120 ng/L; CRP 347 mg/L; procalcitonin 7·42 μg/L; albumin 22 g/L; Platelets 296 × 10^9^	SARS-CoV-2 negative (likely exposure from parent)	7 days; alive

*Adapted from Riphagen et al. (23)*.

*ACA, anterior cerebral artery; CRP, C-reactive protein; HERV, human endogenous retrovirus; HFNC, high-flow nasal canula; HR, heart rate; IVIG, human intravenous immunoglobulin; LOS, length of stay; MCA, middle cerebral artery; MV, mechanical ventilation via endotracheal tube; NIV, non-invasive ventilation; PICU, pediatric intensive care unit; KRT, kidney replacement therapy; SARS-CoV-2, severe acute respiratory syndrome coronavirus 2; VA-ECMO, veno-arterial extracorporeal membrane oxygenation*.

**Figure 2:**
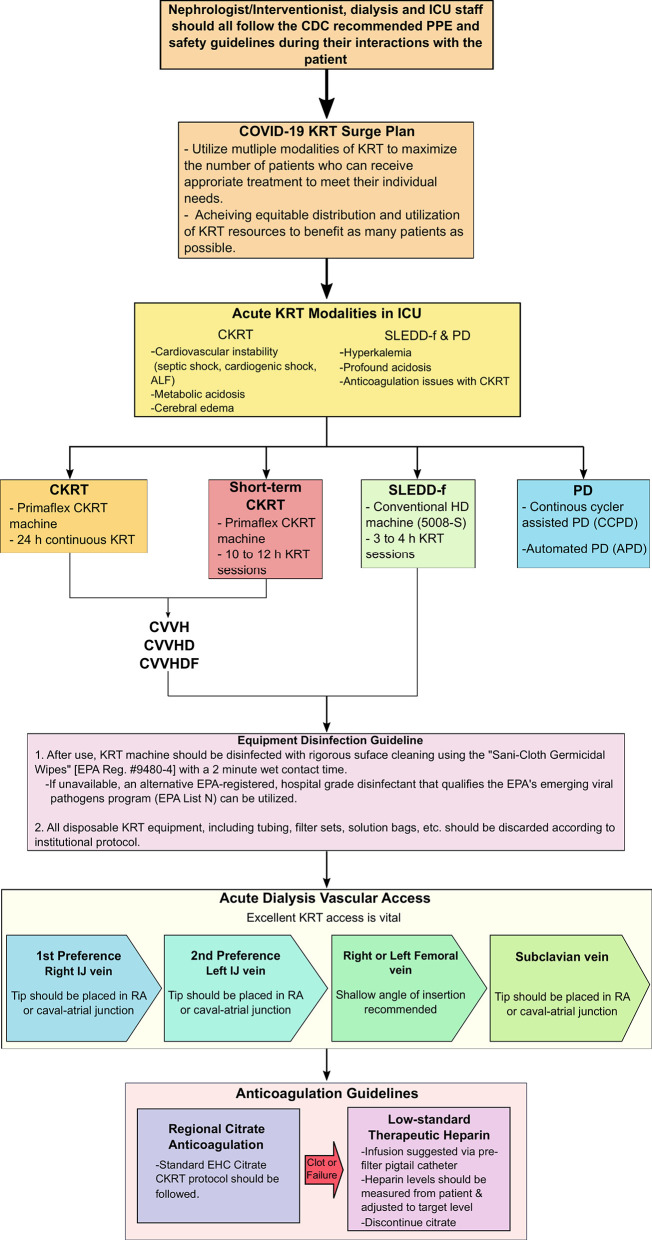
Nephrologist/Interventionist emergency preparedness plan with resource allocation. ALF, acute liver failure; CDC, Centers of Disease Control and Prevention; CKRT, continuous kidney replacement therapy; CVVH, continuous venovenous hemofiltration; CVVHD, continuous venovenous hemodialysis; CVVHDF, continuous venovenous hemodiafiltration; EPA, Environmental Protection Agency; ICU, intensive care unit; IJ, intrajugular vein; KRT, kidney replacement therapy; PD, peritoneal dialysis; PPE, personal protective equipment; RA, right atrium; SLEDD-f, sustained low-efficiency daily diafiltration, UF, ultrafiltration rate.

Until now, this pandemic has not been as detrimental to the pediatric population, however, the world should be prepared if the situation worsens. Thus, we provide a position paper focusing on creating an emergency preparedness plan with resource allocation, specifically if renal conditions worsen in COVID-19 affected children due to the onset of AKI (Figure 2).

## Discussion

Currently, there are no effective pharmacological treatments for COVID-19 patients, however, general, and supportive management via mechanical ventilation and hemodynamic support through extracorporeal therapies can potentially be used to improve overall outcomes in severe pediatric cases.

A recently published editorial by Ronco and colleagues postulated the use of hemofiltration or hemoperfusion containing highly biocompatible sorbents and microporous resins, such as HA330/HA380 cartridges, to potentially provide support to various organs in COVID-19 patients (26). In an animal study by Xu et al., the use of HA330 hemoadsorption in an endotoxin induced ARDS model showed significant improvement in oxygenation, partial improvement in barrier permeability, and reduced inflammation and lung edema (27). In addition, a prospective study of 23 sepsis patients were treated with continuous venovenous hemodiafiltration (CVVHDF) and HA330 hemoperfusion. The investigators reported that all patients showed significant increase in pH and reduction of inflammatory cytokines as indicated by improved levels of C-reactive protein (CRP) (28). However, HA330/HA380 cartridges are not readily available in the United States (US). As an alternate, the CytoSorb® (CytoSorbents Corporation, Monmouth Junction, NJ, USA) adsorber and the oXiris® (Baxter, IL, USA) blood purification set may be utilized to enhance cytokine removal. CytoSorb® is an extracorporeal adsorber which was specifically designed to reduce cytokine storm and various other inflammatory markers (29). CytoSorb was recently approved on April 10, 2020 for emergency use to treat COVID-19 patients (≥18 years of age) with imminent or prominent respiratory failure (30). Similarly, oXiris is believed to reduce endotoxin, cytokine and inflammatory mediator levels associated with COVID-19 and was approved by the FDA on April 23, 2020 for similar indications (patients ≥18 years of age) (31). A comparison between the CytoSorb® and oXiris® filter are shown in Table 3 (29).

**Table 3:** Comparison of various filters available for use in COVID-19 patients requiring KRT.

**Characteristics**	**Filters**	
	**CytoSorb (Cytosorbents)**	**oXiris (Baxter)**
Membrane composition	Polystyrene divinylbenzene co-polymer microporous beads (coated with polyvinylpyrrolidone)	AN69 copolymer covered with polyethyleneimine and unfractionated heparin
Sterilization type	Gamma irradiation	Ethylene oxide
Capability of adsorption	Cytokines	Endotoxin and cytokines
Adsorption mode	Hydrophobic interactions	Ionic interactions-cytokines due to sulfonate groups.-endotoxins due to high PEI concentration on inner part of membrane.
Heparin-covered inner surface	No	Yes

*Adapted from Karkar and Ronco (29)*.

*AN69, acrylonitrile and methalylsulfonate*.

Additionally, hemoperfusion cannot be performed in the US due to lack of resources and thus, we must rely on continuous kidney replacement therapies (CKRT) to provide supportive, rather than replacement therapy in the setting of sepsis and MODS. Since instantaneous monitoring of cytokine levels is not available in COVID-19 patients, CKRT can be utilized to non-selectively clear inflammatory mediators via convection, adsorption, and dispersion. Additionally, CKRT may be able to correct fluid overload, adjust immune stability, and manage solute levels to provide hemodynamic stability in pediatric patients experiencing excessive load and high catabolism (32). In previous studies with ARDS patients, CKRT has demonstrated to reduce extravascular fluid in the lungs, achieve acid-base balance, reduce ventilation pressures, increase the lung oxygenation number, and provide less invasive ventilation of CO_2_ (33, 34). Furthermore, various studies have reported treatment of AKI in COVID-19 patient with KRT (9, 35–37). In a retrospective cohort study by Yang et al., COVID-19 patients requiring invasive mechanical ventilation were treated with CKRT and showed significant reduction in mortality rate in comparison to those treated without CKRT (54.5 vs. 74.6%, *p* = 0.032) (35).

Therefore, we postulate that provision of immediate initiation of preemptive CKRT in cases with progression in symptomatic respiratory insufficiency should be considered. Various studies have reported that, overall, up to 9% of COVID-19 infected patients received CKRT (2, 4–6, 9). A concern, however, is that the virus may be present in the effluent from CKRT according to few studies (38, 39). Though overall, only one percent of infected patients have showed the presence of the virus in the blood; thus, making it unlikely that the effluent would be virulent (39).

In terms of CKRT modality, we propose the use of high flow (HF) CVVHDF in critically ill COVID-19 pediatric patients as HF-CVVHDF is able to boost the non-specific removal of the circulatory cytokine peaks in both the pro- and the anti-inflammatory side in accordance to the “peak concentration hypothesis” (40). Furthermore, a study by Liu et al. demonstrated that high-flow CKRT was significantly more effective in clearance of inflammatory cytokines (such as IL-4, IL-6, TNF-α) due to the increased blood flow in comparison to conventional CKRT (*p* < 0.05) (41). The authors recommended that use of HF-CKRT increased the effective adsorption area of the synthetic membrane leading to higher clearance (41). In a different study, Ghani and colleagues evaluated the efficacy of high-volume hemofiltration (HVHF) in comparison to continuous venovenous hemofiltration (CVVH) in the clearance of excess inflammatory mediators in septic patients (42). The investigators reported that HVHF was significantly more effective in reducing cytokine IL-6 levels (*p* = 0.025) and improving the day 7 Sequential Organ Failure Assessment score in comparison to the CVVH group (42). It has also been demonstrated that convective modalities (such as CVVH and CVVHDF) are superior to diffusive modalities (CVVHD) due to the increased ultrafiltration rate and the higher sieving coefficient of the molecule in the convective mode, which further enhances the effect of cytokine removal (43).

Thus, the Pediatric Continuous Renal Replacement Therapy (PCRRT) registry workgroup suggests high flow CVVHDF at 50 ml/kg/h for 12 h followed by step down CVVHDF at a dose of 25–30 ml/kg/h (Table 4) (44). The provider may be able to incorporate the CytoSorb adsorber or oXiris filter into the CKRT circuit for higher clearance. However, the use of these devices are not FDA approved for this population and thus, the provider will need to obtain a compassionate use eIND (exploratory Investigational New Drug) and take extra caution with the use of these systems. If there is a surge of COVID-19 and CVVHDF is not available, various other CKRT modalities such as CVVH and CVVHD may be employed. If there is a situation where resources are limited and CKRT modalities are not readily available, sustained low-efficiency daily diafiltration (SLEDD-f) or acute PD [specifically, continuous cycler assisted PD (CCPD) or automated PD (APD)] may be utilized (Figure 2).

**Table 4:** Pediatric Continuous Renal Replacement Therapy (PCRRT) registry group suggestions for critically ill, pediatric COVID-19 patients.

•CVVHDF is recommended as the preferred modality as both convection and diffusion allows for removal of bigger molecules which may thus, help in removing inflammatory markers (The rate at which the solute crosses through a membrane is indicated by a number called the sieving. A larger size solute or one with greater affinity to protein binding will have better clearance in CVVHDF than any other CKRT modality). •Preemptive CKRT is suggested if there is progression of respiratory insufficiency, clinical indications of worsening pulmonary edema, and continuing systemic inflammation (high ferritin/CRP and ESR). •High flow CVVHDF is suggested to be performed at a rate of 50 ml/kg/h for the first 12 h followed by step down CVVHDF at a rate of 25–30 ml/kg/h. Alternatively, CVVH, CVVHD, SLEDD-f, or PD should be initiated if resources are not available. •The use of Normocarb bicarbonate-based solutions are recommended. •Circuit clotting in COVID-19 is high due to an increase in procoagulant state and thus, we recommend providing 1/3 of the total replacement fluid pre-filter, another 1/3 should be provided post filter and the remaining replacement fluid should be utilized as dialysate to dilute the circuit. •A higher blood flow rate of 4–5 mL/kg/min is advised to enhance clearance rates of cytokines and reduce the risk of clotting. •The monitoring of electrolyte levels and complete blood count is recommended to be performed every 2 h with high flow CVVHDF and then every 6 h in stepdown CVVHDF. •Nutritional supplementation through adjustment of the replacement fluids and infusion rates are recommended in these patients. •Earlier initiation of KRT is recommended to induce early cytokine clearance and improvement of hemodynamic stability for better outcomes and prevention of multiple organ failure.

*CKRT, continuous kidney replacement therapy; CRP,C-reactive protein;CVVHDF, continuous venovenous hemodiafiltration; ESR, erythrocyte sedimentation rate; PD, peritoneal dialysis; SLEDD-f, sustained low-efficiency daily diafiltration*.

The workgroup also recommends Normocarb bicarbonate-based dialysate solution in pediatric COVID-19 patients. Normocarb bicarbonate-based solutions are the standard of care and are preferred over lactate-based solutions since lactate-based solutions trigger an increase in plasma lactate levels leading to false indications of worsening sepsis and perfusion rates (45–47). In addition, there is a higher risk of circuit clotting in COVID-19 patients due to presence of a hypercoagulable state. Thus, it is recommended that a third of the replacement fluid should be provided pre-filter, a third provided post filter, and the remaining should be utilized as dialysate to dilute the circuit. Anticoagulation should be provided in COVID-19 patients and can be performed with citrate or heparin (48). Citrate allows for localized circuit anticoagulation and is beneficial in patients with active bleeding while unfractionated/low-molecular-weight heparin is able to provide systematic anticoagulation (46–48). However, it is crucial to consider the patient's liver function when deciding on an anticoagulant as COVID-19 patients often have MODS including liver dysfunction and are in a procoagulable state (48). Furthermore, blood flow rate (BFR) is also a crucial aspect of the CKRT prescription and suggested to be maintained at 4–5 mL/kg/min. The higher the BFR, the greater the clearance rate and lessen the risk of clotting (45).

Critically ill patients also often suffer from a significant loss of both macro and micronutrients. Thus, nutritional supplementation during CKRT through the adjustment of replacement fluid composition and infusion rates is suggested to prevent further loss, optimize nutritional status, and recover lean mass with a positive nitrogen balance (49). The PCRRT group further advise the early initiation of KRT in critically ill COVID-19 patients as it has been shown to enhance cytokine clearance, improve PaO_2_/FiO_2_ ratios, mitigate fluid overload, and establish hemodynamic stability earlier, leading to better overall outcomes (33, 50).

**Figure 3:**
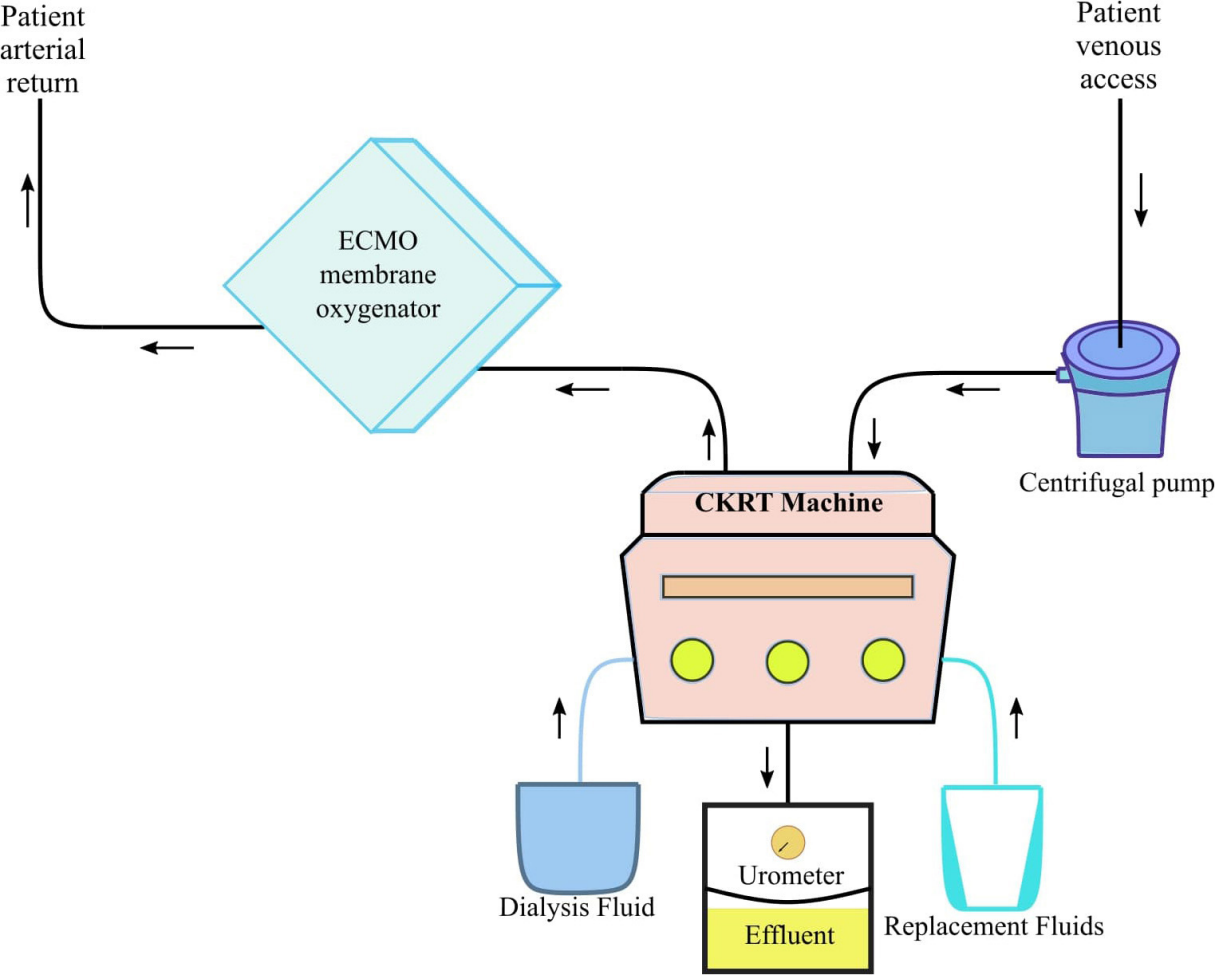
Pediatric ECMO 2.0 with CVVHDF circuit. CKRT, continuous kidney replacement therapy; ECMO, extracorporeal membrane oxygenation. Adapted from Chen et al. (51).

Some critically ill COVID-19 adult patients require mechanical ventilation via extracorporeal membrane oxygenation (ECMO). However, the use of ECMO comes with a risk of potentially amplifying the cytokine activation. Thus, in cases where pediatric patients may require ECMO, we postulate that the CKRT machinery should be incorporated with the ECMO circuit to provide supportive therapy via prevention/reduction of fluid overload and cytokine clearance while providing respiratory sustenance (51). A hemofilter could be placed in line within the ECMO circuit while using intravenous pumps to deliver replacement/dialysate fluid; however, it is not recommended as it can lead to an inaccuracy rate of up to 30% (51). Instead, the PCRRT registry group suggests that the CVVHDF machine should be attached to the ECMO circuit (Figure 3), which would lead to a more efficient delivery of replacement/dialysate fluid dosage with more precise ultrafiltration control (50). In addition, the BFR in the CVVH machine should be independent from the ECMO device and the CKRT machine's venovenous access should be adjusted to tolerate positive pressure since the arterial access of the ECMO will allow for a very low-resistance circuit (47, 51).

In conclusion, COVID-19 is another emerging respiratory virus that has severely challenged the health care system around the world. However, the adult cases have been reported to be more prevalent and severe in comparison to pediatric cases.

Thus, in this position paper, we present an emergency preparedness plan with resource allocation if conditions in the pediatric population worsened dramatically. There is a limitation as this is a position paper grounded on theory of the pathogenesis and anecdotal publication by Ronco et al. (26) and based primarily on adult data and the limited studies available on COVID-19. However, there currently are no effective treatments available and therefore, we suggest the use of high volume CVVHDF in critically ill pediatric COVID-19 patients in the setting of sepsis and MODS. If CVVHDF or the resources required are not available, other KRT modalities, such as CVVHD, SLEDD-f and PD can be utilized. Additionally, incorporation of ECMO circuit with the CVVHDF machinery may improve overall outcomes in COVID-19 patients requiring ventilatory support.

## Data Availability Statement

The original contributions presented in the study are included in the article/supplementary material, further inquiries can be directed to the corresponding author.

## Author Contributions

RR, RC, SS, and TB contributed to the conception and design and wrote sections of the manuscript. All authors contributed to manuscript revision and read and approved the submitted version.

## Conflict of Interest

The authors declare that the research was conducted in the absence of any commercial or financial relationships that could be construed as a potential conflict of interest.
